# Peach DELLA Protein PpeDGYLA Is Not Degraded in the Presence of Active GA and Causes Dwarfism When Overexpressed in Poplar and Arabidopsis

**DOI:** 10.3390/ijms24076789

**Published:** 2023-04-06

**Authors:** Yun Chen, Mengmeng Zhang, Xiaofei Wang, Yun Shao, Xinyue Hu, Jun Cheng, Xianbo Zheng, Bin Tan, Xia Ye, Wei Wang, Jidong Li, Ming Li, Langlang Zhang, Jiancan Feng

**Affiliations:** College of Horticulture, Henan Agricultural University, 95 Wenhua Road, Zhengzhou 450002, China

**Keywords:** *Prunus persica*, DGLLA, DGYLA, gibberellins, dwarfing, evolutionary relationship

## Abstract

Controlling the tree size of fruit species such as peach can reduce the amount of labor and input needed for orchard management. The phytohormone gibberellin (GA) positively regulates tree size by inducing degradation of the GA signaling repressor DELLA. The N-terminal DELLA domain in this protein is critical for its GA-dependent interaction with the GA receptor GID1 and the resulting degradation of the DELLA protein, which allows for growth-promoting GA signaling. In this study, a DELLA family member, PpeDGYLA, contains a DELLA domain but has amino acid changes in three conserved motifs (DELLA into DGYLA, LEQLE into LERLE, and TVHYNP into AVLYNP). In the absence or presence of GA_3_, the PpeDGYLA protein did not interact with PpeGID1c and was stable in 35S-PpeDGYLA peach transgenic callus. The overexpression of *PpeDGYLA* in both polar and Arabidopsis showed an extremely dwarfed phenotype, and these transgenic plants were insensitive to GA_3_ treatment. PpeDGYLA could interact with PpeARF6-1 and -2, supposed growth-promoting factors. It is suggested that the changes in the DELLA domain of PpeDGYLA may, to some extent, account for the severe dwarf phenotype of poplar and Arabidopsis transgenic plants. In addition, our study showed that the DELLA family contained three clades (DELLA-like, DELLA, and DGLLA). *PpeDGYLA* clustered into the DGLLA clade and was expressed in all of the analyzed tissues. These results lay the foundation for the further study of the repression of tree size by PpeDGYLA.

## 1. Introduction

Controlling tree size is critical for reducing the amount of labor and input needed for orchard management [1]. Many fruit growers utilize dwarfing rootstocks to control tree size, which also can reduce the length of the juvenile period, achieve a high production efficiency, and allow for mechanized management [2,3,4]. Otherwise, pruning and spraying growth-regulating agents are usually used for orchard management, especially for peach (*Prunus persica*), which is vigorous in vegetative growth [5]. These horticultural practices used to constrain tree growth are costly in terms of materials, time, and labor. With the rapid development of genetic engineering, the genetic manipulation of tree size through genetic modification may offer a more efficient method than traditional breeding technologies [1]. Therefore, identifying the genes that control tree size will be helpful for peach breeding in the future.

The diterpenoid hormones gibberellins (GAs) play roles in regulating plant growth and development, such as plant height, leaf expansion, grain size, and seed germination [6,7,8,9]. GA levels are precisely controlled by GA biosynthesis and deactivation, in which GA20ox (GA20-oxidase), GA3ox (GA3-oxidase), and GA2ox (GA2-oxidase) are the main genes encoding the regulatory enzymes [10]. In rice, a defective 20-oxidase (a GA biosynthetic enzyme) generates a semidwarf (sd-1) phenotype arising from a deficiency of active GAs in the elongating stem [11]. Mutations of *Atga20ox1* in *Arabidopsis thaliana* and *ZmGA3ox2* (another GA biosynthetic enzyme) in *Zea mays* both result in a dwarf phenotype [12,13]. A poplar mutant displays extremely short internodes and branch length because of hyper-accumulation of mRNA transcripts for *PtaGA2ox1* (a GA catabolic enzyme) and the substantially reduced endogenous GA contents [14].

The GA signaling pathway is mainly comprised of the GA receptor GA-INSENSITIVE DWARF1 (GID1), GA signaling repressors (DELLAs), and the F-box proteins [15,16,17]. The complex of GA-GID1 can enhance the interaction between GID1 and DELLA, and these interactions cause rapid degradation of DELLAs via the 26S proteasome pathway [18]. GA promotes plant growth mainly by inducing the degradation of the DELLA protein, which is well documented to be a growth-inhibiting protein. DELLA functions as a repressor of GA signaling by interacting with growth-promoting transcription factors/regulators. DELLA inhibits hypocotyl elongation in Arabidopsis through physically interacting with phytochrome-interacting factors 3 and -4 (PIF3 and PIF4), and this interaction prevents the expression of *PIF3/PIF4* target genes [19,20]. The auxin-response factor ARF6, the brassinosteroid-signaling transcription factor BZR1, and PIF4 interact with each other and cooperatively regulate plant growth. RGA (DELLA) binds directly to them to block their DNA-binding activities, but this DELLA interaction is inactivated by GA [21].

DELLA belongs to a subgroup of the GRAS transcription factors and has a conserved DELLA domain in its N-terminal and a conserved GRAS domain in its C-terminal [22]. The DELLA domain is characterized by three motifs, DELLA, LEQLE, and TVHYNP, which play roles in the GA-dependent interaction with the GA receptor GID1 and the GA-induced degradation of DELLA protein [16,23]. The three motifs involved in the interaction of DELLA and GID1 were analyzed by modeling the structure of the putative complexes between AtGID1a and DELLAs from species of different land plants and by yeast two-hybrid assays [24]. Deletion of the DELLA and TVHYNP motifs in rice abolished the GA-dependent interaction between SLR1 and GID1 and reduced the transcriptional activation of SLR1 [23,25]. In the DELLA motif, a change in the fourth amino acid leucine (L) to histidine (H) led to a gain-of-function phenotype (dwarf) in grapevine [26]. This suggests that some conserved amino acids in these three motifs of the DELLA domain are important for DELLA-GID1 interaction. However, the mechanisms underlying the perception of GA signals by DELLA-like protein in peach have not been reported. To date, two DELLA proteins and one DELLA-like protein have been identified in peach, but the function of DELLA-like remains poorly understood.

Our previous study identified a peach DELLA/-like family member, PpeDGYLA, which has several amino acid changes in the DELLA domain [27]. In this study, we analyzed the structural features and transcription levels of *PpeDGYLA* in different tissues of peach. The gene function of *PpeDGYLA* was analyzed by overexpressing it in poplar and Arabidopsis. In addition, the stability of the PpeDGYLA protein was analyzed by overexpressing it in peach calli. Two candidate proteins that interact with PpeDGYLA were identified by Y2H assay. Finally, the evolutionary relationship of PpeDGYLA with the other two peach DELLA proteins was analyzed by constructing a phylogenetic tree.

## 2. Results

### 2.1. PpeDGYLA Carries an Altered DELLA Motif of DGYLA

**Figure 1:**
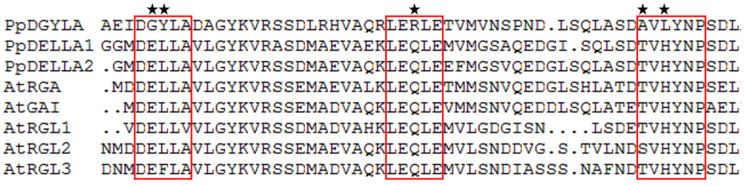
DELLA domains of Arabidopsis and peach DELLA family members. Three conserved motifs (DELLA, LEQLE, and TVHYN) in the DELLA domain are highlighted by red boxes. In PpeDGYLA, the three motifs are changed into DGYLA, LERLE, and AVLYNP and the non-canonical amino acids are indicated with black stars.

In peach, a DELLA family member (*Prupe.7G236100*) was found to encode a deduced protein of 537 amino acid residues. Multiple sequence alignment showed that this DELLA family member contained a DELLA domain that showed some differences compared with other DELLA proteins (Figure 1). The DELLA domain is comprised of three conserved motifs (DELLA, LEQLE, and TVHYNP). In this DELLA family member, the three motifs were changed into DGYLA, LERLE, and AVLYNP (Figure 1). Therefore, this member was named *PpeDGYLA*. Sequence similarity analysis was conducted using three peach DELLA members (PpeDGYLA and PpeDELLA1/-2) and five Arabidopsis DELLA members (RGA, GAI, and RGL1/-2/-3). PpeDGYLA showed a range of 39.94–44.96 % sequence similarity when aligned with the seven DELLA proteins (PpeDELLA1/-2, AtRGA, AtGAI, and AtRGL1/-2/-3), while the seven DELLA proteins had a range of 50.55–73.59 % when aligned with each other (Table 1). This suggests that PpeDGYLA had a relative low sequence similarity with other DELLA proteins.

The theoretical isoelectric point (pI) of PpeDGYLA was 5.58 with a calculated molecular mass of 59.16 kDa. PpeDGYLA was predicted to be an unstable hydrophobic protein based on the instability index (II) and the grand average of hydropathicity (GRAVY) (Table S2), and was comprised of an alpha helix, an extended strand, and a random coil with percentages of 46.00 %, 12.48 %, and 41.53 %, respectively (Table S2 and Figure S1).

### 2.2. PpeDGYLA Was More Stable than PpeDELLA1 after GA_3_ Treatment

The DELLA domain mediates the GA-dependent interaction with the GA receptor GID1 and its own GA-induced degradation [16,23]. Multiple sequence alignment showed that several conserved amino acids were changed in the DELLA domain of PpeDGYLA. Therefore, we hypothesized that the GA-dependent interaction with GID1 and the degradation of PpeDGYLA were affected due to the changes in its DELLA domain. Our previous study showed that the GA receptor PpeGID1c could interact with PpeDELLA1 in the presence of active GA, which then induced the degradation of PpeDELLA1 and increased the plant height of peach [5].

**Figure 2:**
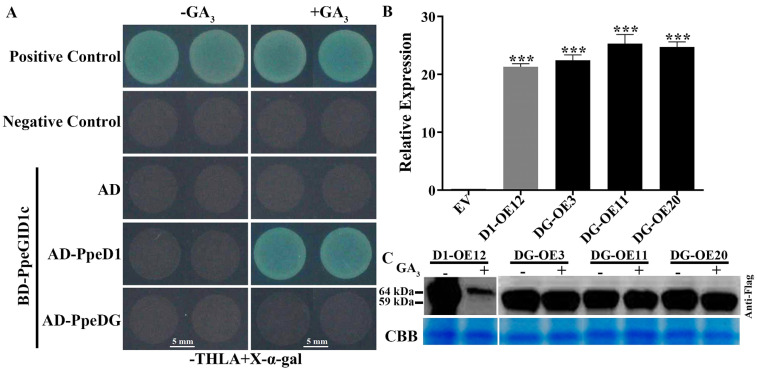
PpeDGYLA was stable in the presence of active GA. (**A**) Y2H assays with or without supplemental GA_3_. Transformed yeast cells are grown on SD-Trp/-Leu/-His/-Ade medium containing 20 mg/L X-α-gal. Yeast containing BD-PpeGID1c and AD-PpeDELLA1 (AD-PpeD1) grows normally and turns blue in the presence of GA_3_, while yeast containing BD-PpeGID1c and AD-PpeDGYLA (AD-PpeDG) does not grow in the presence or absence of GA_3_. BD-53+AD-T7 and BD-Lam+AD-Lam are used as the positive control and negative control, respectively. The experiment is replicated three times. -GA_3_, without gibberellin; +GA_3_, with 10 μM gibberellin. Scale bars = 5 mm (**B**) 35S-PpeDELLA1-flag and 35S-PpeDGYLA-flag are delivered into the peach leaf callus. qRT-PCR analysis of PpeDELLA1 (D1-OE) and PpeDGYLA (DG-OE) transcript levels in transgenic callus. Three *PpeDGYLA* lines are obtained. Asterisks denote significant differences compared with EV (***, *p* < 0.001). (C) Western blots showing the levels of PpeDELLA1 or PpeDGYLA protein in the transgenic callus overexpressing *PpeDELLA1*-*flag* or *PpeDGYLA*-*flag* after treatment with 100 μM GA_3_ (+) or mock (−) solution. Coomassie brilliant blue staining the Rubisco small subunit (RbcS) protein is used as a loading control.

In this study, we analyzed the interaction between PpeDGYLA and PpeGID1c and the protein level of PpeDGYLA under GA_3_ treatment. In the Y2H assay, yeast containing BD-PpeGID1c and AD-PpeDELLA1 could not grow on SD/-THLA+X-α-gal without GA_3_, but grew normally in the presence of GA_3_. Switching out the DELLA protein, yeast containing BD-PpeGID1c and AD-PpeDGYLA could not grow in the presence or absence of GA_3_ (Figure 2A). This suggests that the GA-dependent interaction between PpeGID1c and PpeDGYLA was interrupted.

In peach leaf callus carrying *35S*::*PpeDELLA1*-*flag* and *35S*::*PpeDGYLA*-*flag* (Figure 2B), the protein levels of PpeDELLA1-flag and PpeDGYLA-flag were analyzed using Western blot assay. In the absence of GA_3_, PpeDELLA1-flag and PpeDGYLA-flag were both detected by an anti-flag antibody. In the presence of GA_3_, the PpeDELLA1-flag protein level decreased significantly, while the protein content of PpeDGYLA-flag showed no change (Figure 2C). These results suggest that PpeDGYLA could not be degraded in the same way as PpeDELLA1 with GA_3_ treatment.

### 2.3. PpeDGYLA Overexpression Results in a Severe Dwarf Phenotype in Poplar and Arabidopsis

**Figure 3:**
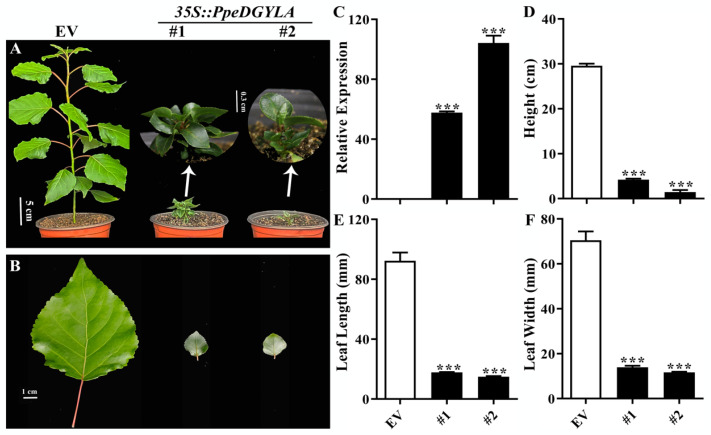
Phenotypes of poplar overexpressing *PpeDGYLA*. (**A**) Six-week-old empty vector (EV) and *PpeDGYLA-OE* transgenic plants cultivated under long-day growth conditions. Scale bars = 5 cm or 0.3 cm for the two insets. (**B**) Leaves of EV and transgenic plants shown in A. Scale bars = 1 cm. (**C**) Transcript levels of *PpeDGYLA* in EV and *PpeDGYLA-OE* transgenic lines. (**D**) The height of 6-week-old plants. (**E**,**F**) The length and width of the fifth and sixth leaves shown in A. Asterisks denote significant differences (***, *p* < 0.001). Error bars represent SD of plants in (**D**–**F**) (*n* = 12).

The high stability of PpeDGYLA in the presence of active GA meant that *PpeDGYLA* overexpression might have resulted in a more severe dwarf phenotype than when *PpeDELLA1* was overexpressed. Because it is very hard to conduct stable transformation of peach, *35S*-*PpeDGYLA* was delivered into poplar, and two transgenic lines highly expressing *PpeDGYLA* were obtained (Figure 3C). An empty vector (EV) construct was used as the control. The two transgenic lines showed an extremely dwarfed phenotype compared with the control (Figure 3A). The leaves of the two transgenic lines were smaller than the control (Figure 3B). These transgenic plants were transplanted from the WPM medium to soil, and were cultured for another 6 weeks before their height and leaf size were measured (Figure 3D−F). The plant height of the two transgenic lines were 15 % and 4 % of the control, respectively.

**Figure 4:**
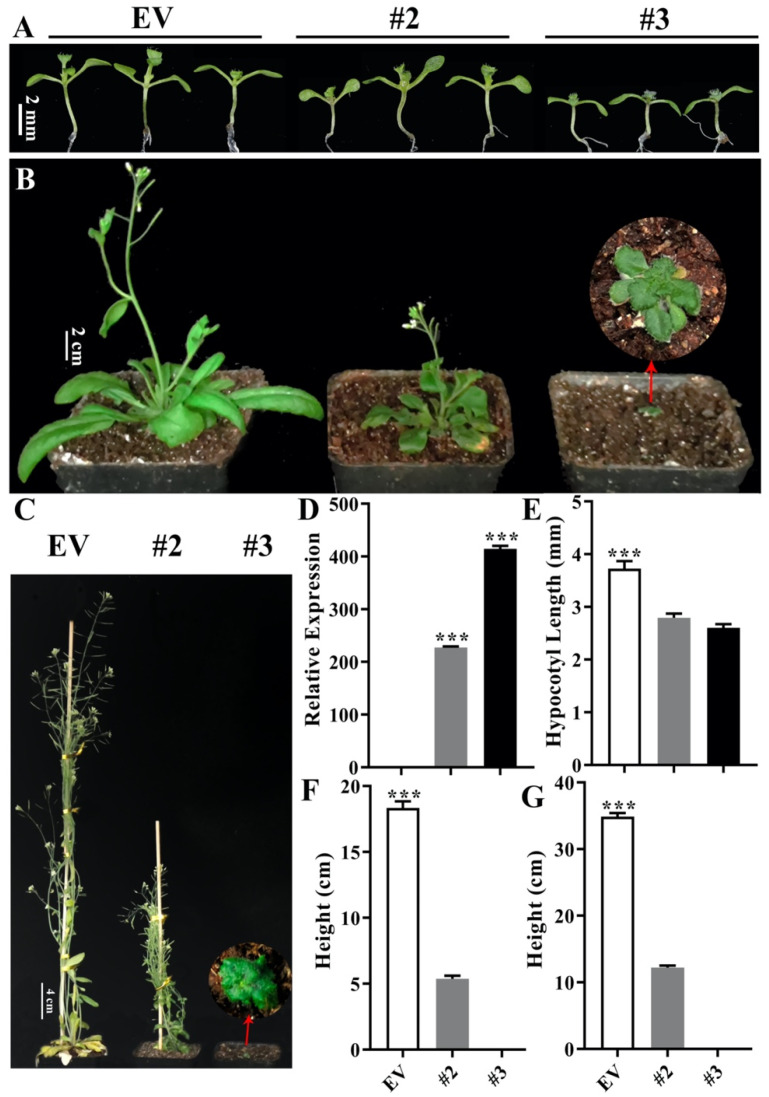
Phenotypes of Arabidopsis overexpressing *PpeDGYLA*. (**A**–**C**) Phenotypes of 7-d, 25-d and 35-d-old OE and EV plants cultivated under long-day growth conditions. Scale bars = 2 mm/2 cm/4 cm. (**D**) The expression level of *PpeDGYLA* in transgenic OE and EV lines. (**E**) The hypocotyl length of seedlings shown in (**A**). (**F**,**G**) The height of seedlings shown in (**B**,**C**), respectively. Asterisks denote significant differences (***, *p* < 0.001). Error bars represent SD of plants in (**E**–**G**) (*n* = 12).

In Arabidopsis, *35S*-*PpeDGYLA* T1 generation plants were used to analyze the plant height. Similar results were obtained in these Arabidopsis *PpeDGYLA* transgenic lines. The hypocotyl length and the plant height of the 25-day-old and 35-day-old plants were photographed and measured (Figure 4). Two Arabidopsis *PpeDGYLA* transgenic lines (line 2 and 3) showed a dwarfing phenotype, with line #3 having an extremely dwarfed phenotype (Figure 4). Because of the extremely dwarfed phenotype of line #3, it was difficult to obtain the seeds and T1 generation plants were used to analyze the phenotype. These results demonstrate that overexpressing *PpeDGYLA* severely inhibited the growth of transgenic plants.

### 2.4. Poplar and Arabidopsis PpeDGYLA-OE Transgenic Plants Were Insensitive to GA_3_ Treatment

**Figure 5:**
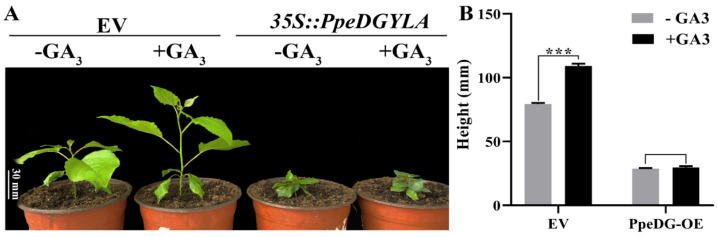
Poplar overexpressing *PpeDGYLA* exhibited a GA-insensitive phenotype. (**A**) Phenotypes of EV and *PpeDGYLA*-overexpressing (OE) plants 7 d after treatment with 100 μM GA_3_ or a mock solution. Scale bars = 30 mm. (**B**) The height of the EV and OE plants after treatment with 100 μM GA_3_ or a mock solution. The values are means ± SD (*n* = 12; ***, *p* < 0.001).

**Figure 6:**
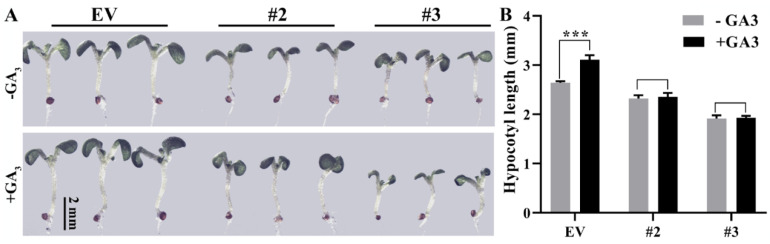
Arabidopsis overexpressing *PpeDGYLA* exhibited a GA-insensitive phenotype. (**A**) Phenotypes of empty vector (EV) and *PpeDGYLA*-overexpressing (OE) Arabidopsis plants after 3 d of treatment with 1 μM GA_3_ or a mock solution. Scale bars = 2 mm. (**B**) The hypocotyl length of the EV and OE transgenic seedlings after being treated with 1 μM GA_3_ or a mock solution. The values are means ± SD (*n* = 18; ***, *p* < 0.001).

Our results demonstrated that GA_3_ treatment could not induce the degradation of PpeDGYLA. Therefore, we speculated that poplar and Arabidopsis PpeDGYLA transgenic plants might be insensitive to GA treatment. We examined the effect of bioactive GA (GA_3_) on plant height in *PpeDGYLA*-OE poplar and Arabidopsis plants. After treatment with 100 µM GA_3_ for 7 d, the poplar EV plants were significantly taller than the untreated controls (0 µM GA_3_), while *PpeDGYLA*-OE plants showed no obvious change in height (Figure 5A,B). The same results were observed in *PpeDGYLA*-OE Arabidopsis after treatment with 1 µM GA_3_ for 3 d. After GA_3_ treatment, Arabidopsis EV plants had a longer hypocotyl than the control, but *PpeDGYLA*-OE plants had no obvious change in hypocotyl length (Figure 6A,B). These results suggest that poplar and Arabidopsis *PpeDGYLA* transgenic plants were insensitive to GA_3_ treatment.

### 2.5. PpeDGYLA Interacts with PpeARF6-1/-2

**Figure 7:**
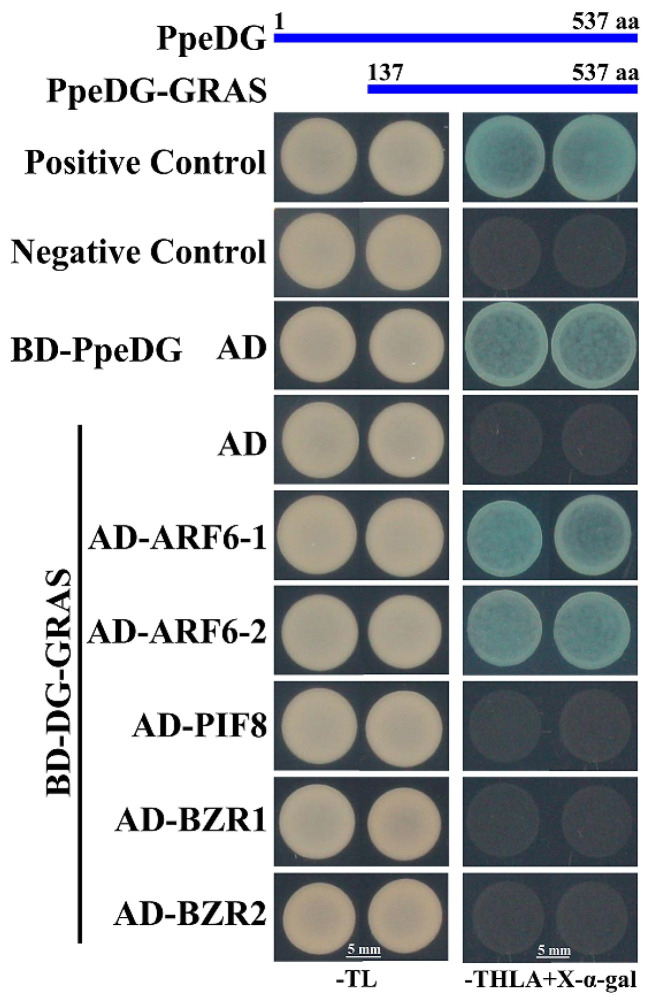
Yeast two-hybrid assays showing the interactions between PpeDGYLA and PpeARF6-1 and -2, PpePIF8, or PpeBZR1 and -2. The combinations of BD-53+AD-T7 and BD-Lam+AD-Lam are used as the positive control and negative control, respectively. Transformed yeast cells are grown on SD-Trp/-Leu (−TL) and SD-Trp/-Leu/-His/-Ade medium (−TLHA) containing 20 mg/L X-α-gal. This experiment is replicated three times. Upper: Diagram of protein structures of PpeDGYLA and its truncation (GRAS) used for the yeast two-hybrid assay.

Numerous studies have demonstrated that DELLA proteins repress plant growth mainly by blocking the DNA-binding capacity of growth-promoting factors, such as ARF6, PIFs, and BZR1 [21,28,29]. In this study, we investigated the interactions of PpeDGYLA with PpeARF6, PpePIF8, and PpeBZR1 using Y2H assays. Our previous study showed that PpePIF8 played a positive role in the growth of peach. The homologous genes of ARF6 and BZR1 were identified by constructing phylogenetic trees (Figure S2). Two genes related to ARF6 and two related to BZR1 were identified from the peach genome. The full-length PpeDGYLA protein had a transcriptional activation activity, and PpeDGYLA-GRAS (DG-GRAS) was used for Y2H assays (Figure 7). The results showed that PpeDGYLA-GRAS interacted with PpeARF6-1 and -2 in yeast (Figure 7).

### 2.6. The Evolutionary Relationship between PpeDGYLA and Other DELLA Members

In this study, our results suggest that PpeDGYLA may be different from the other two peach DELLA family members. Therefore, a phylogenetic tree was constructed using the DELLA members from different land plant taxa, including bryophytes, lycophytes, gymnosperms, and angiosperms. All DELLA members were divided into three types (Figure S3). DELLA members from bryophytes and lycophytes clustered into Type 1 and were named DELLA-Like (DL), while DELLA members from gymnosperms and angiosperms were clustered into Type 2 and 3. Type 2 contained DELLA members that mostly contained a DELLA domain starting with the DELLA (DE) amino acid sequence, while Type 3 contained DELLA members that mostly contain DELLA domains starting with DGLLA (DG). PpeDGYLA was clustered into Type 3.

The types of DELLA proteins in each species were analyzed according to the phylogenetic tree (Table 2). Bryophytes and lycophytes contained just Type 1 DELLA proteins. All of the analyzed gymnosperms and angiosperms contained DELLA members from Type 2. Among the analyzed gymnosperms and angiosperms, there were 12 species that lost the DELLA proteins from Type 3 (Table 2), including the model species Arabidopsis and poplar.

### 2.7. Transcript Levels of PpeDELLA in Different Tissues

**Figure 8:**
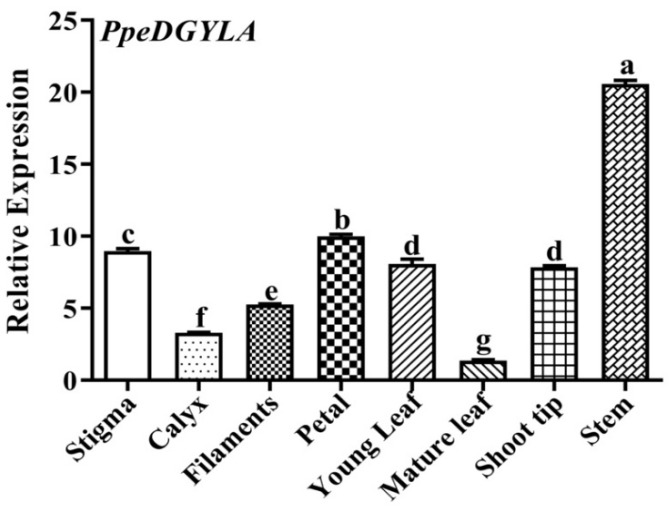
Tissue-specific expression of *PpeDGYLA*. Eight tissues were collected from 6-year-old trees of the peach cultivar ‘QiuMiHong’ (QMH). The error bars indicate the mean of three independent experiment replicates ± standard deviation (SD). Different letters indicate significant differences (*p* < 0.05) according to Duncan’s test by ANOVA.

To analyze the tissue-specific expression of *PpeDGYLA*, eight tissues (different floral organs, leaves, shoot tips, and stems) were collected from peach ‘QMH’. qRT-PCR analysis showed that *PpeDGYLA* was expressed in all eight tissues (Figure 8). Among these tissues, *PpeDGYLA* transcript levels were highest in the stems and lowest in mature leaves.

The transcript levels of the three peach DELLA family members were compared using the stem, flower, and fruit transcriptome data in the NCBI database (PRJNA723104). The information for these samples was reported in our previous study [30]. The three peach DELLA members were expressed in these samples, and *PpeDGYLA* showed a higher transcript level in all of the analyzed samples compared with *PpeDELLA1* (Figure S4). This implied, to some extent, that *PpeDGYLA* plays an indispensable function in the plant growth and development of peach.

## 3. Discussion

### 3.1. Amino Acid Changes in the DELLA Domain of PpeDGYLA May to Some Extent Account for the Severe Dwarf Phenotype of Poplar and Arabidopsis Transgenic Plants

GA promotes plant growth by inducing degradation of the DELLA protein. DELLA is a growth repressor that interacts with growth-promoting factors such as PIF proteins, ARF6 and BZR1 [19,20,21,28]. The DELLA protein is comprised of two domains, the DELLA domain and the GRAS domain. The GRAS domain is responsible for the interaction between DELLA and these growth-promoting factors [31,32,33,34]. PpeDGYLA is highly homologous to other DELLA proteins and contains a conserved GRAS domain and several key alterations with the DELLA domain. Our results demonstrate that overexpressing *PpeDGYLA* in poplar and Arabidopsis resulted in a dwarf phenotype. A similar result was reported in rice, in which overexpressing *SLRL1*, a member of the Type 3/DGLLA clade, induced a dwarf phenotype [35]. Moreover, PpeDGYLA could interact with the ARF6 orthologous protein PpeARF6-1/-2 through its GRAS domain. These results suggest that *PpeDGYLA* is similar to *DELLA* and plays a negative role in plant growth.

Our previous study showed that overexpressing *PpeDELLA1/-2* (two peach *DELLA* genes) just slightly inhibited plant height (semi-dwarf) in Arabidopsis [27]. In this study, our results show that overexpressing *PpeDGYLA* resulted in a severe dwarf phenotype in both Arabidopsis and poplar. We hypothesized that the amino acid residue differences within the DELLA domain were responsible for the discrepancy in plant height between the *PpeDELLA1/-2* and *PpeDGYLA* transgenic plants. Our results show that the three conserved motifs (DELLA, LEQLE, and TVHYNP) in PpeDGYLA were changed into DGYLA, LERLE, and AVLYNP. Studies have shown that these three motifs help to mediate the GA-dependent interaction with between the GA receptor GID1 and the GA-induced degradation of DELLA [16,23]. In this study, our results show that the PpeDELLA1 protein, but not PpeDGYLA, could interact with PpeGID1c and be degraded after GA_3_ treatment. Moreover, poplar and Arabidopsis plants ectopically overexpressing *PpeDGYLA* were insensitive to GA_3_ treatment. In Arabidopsis, a 17-amino acid deletion within the DELLA domains of GAI and RGA both caused a GA-unresponsive, severe dwarf phenotype [36]. On the contrary, the dwarf phenotype caused by overexpressing a normal *RGA* gene could be recovered by GA_3_ treatment [36]. Interestingly, a single amino acid mutation in the DELLA motif of a grape DELLA protein resulted in a dwarfing phenotype [26]. From apple (*Malus hupehensis* Redh. var. pingyiensis), the overexpression of *Mhgai1* (a mutation of a single amino acid in the DELLA, mutated to VELLA) or *Mhgai2* (DELLA mutated to DELHA) in tomato exhibited GA-insensitive plants with smaller statures and smaller fruits and seeds [37,38]. Together, these results suggest that changes in the DELLA domain of PpeDGYLA stabilized the protein in the presence of active GA and resulted in a GA-insensitive and severe dwarf phenotype.

### 3.2. DGLLA Is an Important Clade in the DELLA Family

Evolutionary analysis showed that ancestral DELLA evolved into three types of DELLA: DELLA-1, -2, and -3 [24]. Our results show that the DGLLA clade was identical to DELLA3. The DGLLA clade family was retained in vascular plants (lycophytes, gymnosperm, monocots, and eudicot), except for the bryophytes [39]. In this study, PpeDGYLA, one member of the DGLLA clade, was expressed in all of the analyzed tissues and had a higher expression level than PpeDELLA1 in the stems, fruits, and flowers. Moreover, poplar and Arabidopsis plant overexpression PpeDGYLA showed a GA-insensitive dwarf phenotype, which is different from the dwarfing phenotype caused by the overexpression of genes in the DELLA1 clade [36]. This implies that DGLLA is an important clade in the DELLA family and may be different from the DELLA1/2 clade in the gene function.

It is worth noting that not all species of eudicots and monocots contained the DGLLA clade. Hernández-García et al. [24] demonstrated that DELLA3/DGLLA was absent in tomato. In this study, our results show that DGLLA members were absent in 12 species among the analyzed eudicots and monocots. This seems to mean that DGLLA was dispensable for these species. However, Li et al. [40] reported two DELLA-like proteins (SlGLD1 and -2) that lacked the DELLA domain and only contained the GRAS domain. Overexpressing *SlGLD1* in tomato caused a severe dwarf phenotype. In addition, a common characteristic of DGLLA members from the grass family is a lack of DELLA domain [41]. Together, it was speculated that the function of DGLLA could be carried out by some DELLA-like proteins that lack the DELLA domain.

It is regrettable that the specific roles of DGLLA in plant development and growth are not very clear. Our results show that DGLLA is absent in the well-known model plant Arabidopsis and in tomato and poplar. Therefore, there has been no study until now reporting on the phenotype of plants in which DGLLA is knocked out.

### 3.3. It Is Meaningful to Compare the DELLA Domain between PpeDELLA1/-2 and PpeDGYLA

In an agricultural setting, controlling the tree size at the cultivar level can reduce the amount of labor and input needed for orchard management. Plant growth could be regulated by changing the strength of the interaction between GID1 and SLR1. Eight *gid1* mutants with mutations in its coding sequence showed a series of dwarfing phenotypes, with different plant height, in rice [23]. The DELLA domain in the N-terminal portion of the protein was important for the interaction of GID1c and DELLA. The DGLLA member OsSLRL1 had no DELLA domain and resulted in a dwarf phenotype when overexpressed in rice, while overexpressing SLR1-SLRL1 (a fusion protein of the N-terminal portion of SLR1 with SLRL1) resulted in a severe dwarf phenotype of rice [35]. In this study, our results show that several conserved amino acids were changed in the DELLA domain of PpeDGYLA, which abolished the PpeGID1c interacting activity under GA_3_ treatment. This implied that the change in these conserved amino acids might have accounted for the interruption of the interaction between PpeDGYLA and PpeGID1c.

## 4. Materials and Methods

### 4.1. Plant Materials and Growth Conditions

The peach cultivar ‘QiuMiHong’ (QMH) was obtained from the Fruit Tree Germplasm Repository of Henan Agricultural University (Zhengzhou, Henan Province, China). The different tissues collected from ‘QMH’ were used to analyze the transcript levels of *PpeDGYLA*. Stigma, calyx, filaments, and petal were from flowers at full bloom from 6-year-old ‘QMH’ trees. Young leaves, mature leaves, shoot tips, and stems were collected according to Chen et al. [29].

*Arabidopsis thaliana* ecotype Columbia-0 (Col-0) plants used for *PpeDGYLA* transformation were grown in a culture room (22 ± 1 ºC; 16/8 h light/darkness; light intensity: 150 µmol m^−2^ s^−1^).

The progeny of *Populus deltoides* × *P. euramericana* cv ‘Nanlin895′ was used for *PpeDGYLA* transformation and cultured in Woody Plant Medium (WPM) under a 16/8 h light/darkness at 23 ± 1 °C.

### 4.2. Gene Cloning, Sequence Analysis, and Phylogenetic Tree Construction

The total RNA was extracted from leaf samples of ‘QMH’ trees using an RNA isolation kit (Huayueyang Biotech, Beijing, China) according to the manufacturer’s instructions. cDNA was synthesized from RNA using a HiScript III 1^st^ Strand cDNA Synthesis Kit (+gDNA wiper) (Vazyme, Nanjing, China). The coding regions of the *PpeDGYLA*, *PpeDELLLA1*, *PpeARF6-1*/*-2*, *PpePIF8,* and *PpeBZR1*/*-2* genes were amplified from the cDNA using specific primers (Table S1). The resulting PCR amplicons were purified and a vector was used for construction.

The physical and chemical properties of the PpeDGYLA protein were predicted using the Expasy protparam tool (https://web.expasy.org/protparam/) (accessed on 3 December 2022). The secondary structure of PpeDGYLA was predicted using PSIPRED (http://bioinf.cs.ucl.ac.uk/psipred/) (accessed on 3 December 2022) and GORIV (http://npsa-pbil.ibcp.fr/cgi-bin/npsa_automat.pl?page=npsa_gor4.html) (accessed on 3 December 2022).

The amino acid sequences of DELLA-like proteins were downloaded from the *Arabidopsis database* (TAIR) (https://www.arabidopsis.org/index.jsp) (Accessed on 5 December 2022). All of the protein sequences of different species were downloaded from Phytozome 12 (https://phytozome.jgi.doe.gov/pz/portal.html#) (Accessed on 5 December 2022) or the National Center for Biotechnology Information (https://www.ncbi.nlm.nih.gov/) (Accessed on 5 December 2022) for the construction of the phylogenetic tree. Multiple sequence alignment was performed using DNAMAN. The phylogenetic tree was constructed using the neighbor-joining method of MEGA 6.0 with the default parameters and setting as follows: Bootstrap method, 1000 replicates, and the Poisson model.

### 4.3. Transformation of Poplar and Arabidopsis

The CDS fragment of *PpeDGYLA* was cloned into the pSAK277 (with 35S promoter) vector to generate a *PpeDGYLA*-overexpression construct. The recombinant plasmid and empty vector were transformed into wild-type Arabidopsis using the floral dip method [42]. Transgenic Arabidopsis plants were selected and confirmed by qRT-PCR using specific primers of the *PpeDGYLA* gene. The phenotypic analyses used T1 transgenic plants. The length of hypocotyl and the height of the plants from different *PpeDGYLA* and SAK277 transgenic lines (EV) were measured and photographed at the age of 7 d, 25 d, and 35 d.

Poplar plants were transformed by infecting leaf discs with *Agrobacterium tumefaciens* cultures carrying *pSAK277*-*PpeDGYLA* [43,44,45]. After co-culturing with Agrobacteria on WPM containing 20 mg/L acetosyringone for 3 d under darkness, the infected leaf discs were transferred onto fresh medium containing 0.5 mg/L kinetin (KT), 0.75 mg/L 2,4-Dichlorophenoxyacetic acid (2,4-D), 400 mg/L cephalosporin, and 50 mg/L kanamycin and 0.7 % (*w*/*v*) agar to induce callus. After culturing for about 3 weeks in the dark, the leaf discs with calli were transferred to adventitious-bud-inducing medium containing 0.02 mg/L thidiazuron (TDZ), 400 mg/L cephalosporin, and 50 mg/L kanamycin and 0.7% (*w*/*v*) agar. Regenerated shoots were placed in a rooting medium containing 400 mg/L cephalosporin and 50 mg/L kanamycin and 0.7 % (*w*/*v*) agar. The rooted plantlets were characterized by qRT-PCR. The positive transgenic plants were acclimatized in pots placed in an artificial climate chamber (NingBo Ledeng Instrument Manufacturing, NingBo, China; 16/8 h light/darkness, 25 ± 1 °C) for 2 weeks and then grown in the culture room (temperature: 25 ± 1 °C; photoperiod: 16/8 h light/darkness). Two transgenic lines (#1, #2) were selected by qRT-PCR for the phenotyping assays. The length of the leaves, the width of the leaves, and the height of the plants from different transgenic lines were measured and photographed at the age of 6 weeks. More than 12 seedlings were used for statistical analysis.

### 4.4. Hormone Treatment

For the poplar experiment, the positive transgenic and empty vector (EV) plants were transplanted into a growth media (peat: vermiculite: perlite = 3:1:1) and grown in the culture room for 2 weeks. Next, the plants were sprayed with 100 μM GA_3_ (Solarbio, Beijing, China) or a mock solution three times within 24 h. Seven days after treatment, the phenotype was photographed and the height of the EV and transgenic plants was measured. At least 12 plants were used for every treatment, and the entire experiment was replicated three times.

For the Arabidopsis experiment, *PpeDGYLA-*overexpressing and EV transgenic Arabidopsis were germinated on MS (Murashige and Skoog, Sigma, Germany) medium for 5 d in an artificial climate chamber (16/8 h light/darkness, 22 ± 1 °C). One group was transferred to new MS medium containing 1 μM GA_3_, another group was transferred to new MS medium containing mock solution. After 3 d of treatment, the phenotypes were photographed, and the hypocotyl lengths of the EV and transgenic seedlings were measured. At least 18 plants were used for every treatment, and the entire experiment was replicated three times. All data were analyzed by ANOVA, Duncan’s multiple range tests (at *p* < 0.05), and Student’s *t*-test using IBM SPSS Statistics 20 (SPSS, Chicago, IL, USA).

### 4.5. Yeast Two-Hybrid (Y2H) Assays

The coding sequences of *PpeGID1c* were cloned into pGBKT7, while those of *PpeDGYLA* and *PpeDELLLA1* were fused into pGADT7. Y2H assays were carried out as reported [29]. In brief, three pairs of constructs, namely BD: PpeGID1c+AD:Lam, BD:PpeGID1c+AD:PpeDELLA1, and BD:PpeGID1c+ AD: PpeDGYLA, were co-transformed into Y2HGold. BD-53+AD-T7 and BD-Lam+AD-Lam were used as the positive control and negative control, respectively. Each interaction was tested on SD/-Trp/-His/-Leu/-Ade (−THLA) (Clontech, Mountain View, CA, USA) with X-α-gal medium in the absence and presence of 10 μM GA_3_.

The full-length coding regions of *PpeDGYLA* and *PpeDGYLA*-GRAS (a deletion of 408 bp in the N-terminal) were cloned into pGBKT7, while that of *PpeARF6-1*/*-2*, *PpePIF8,* and *PpeBZR1*/*-2* were fused into pGADT7. Y2H assays were carried out as above. Transformed yeast cells were grown on SD/-Trp/-Leu (−TL) (Clontech, Mountain View, CA, USA) and SD/-Trp/-His/-Leu/-Ade (−THLA) with X-α-gal media. All of the experiments were replicated three times. All of the primers used for cloning in this study are shown in Table S1.

### 4.6. Analyzing the Stability of PpeDGYLA Protein

The *PpeDGYLA* sequence was amplified from cDNA of ‘QMH’ and cloned into the pSAK277-flag vector to produce the *PpeDGYLA*-flag overexpression construct. Shoots from 2-year-old ‘QMH’ peach trees were used for the transformation. The shoots were surface sterilized in 75 % ethanol for 1 min and then in 1% sodium hypochlorite for 10 min, followed by washing with sterile distilled water five or six times. The *PpeDGYLA*-flag-overexpression (OE) peach callus lines were obtained from these shoots according to Xu et al. [46]. The calli were characterized by qRT-PCR.

For GA_3_ treatment, the freshly sub-cultured transgenic and control peach calli were cultured on MS agar medium containing 0 µM or 100 µM GA_3_ for 48 h. For Western blot, the protein of the treated and control peach calli was extracted using the TCA-acetone method [47]. The protein was detected by immunoblot using anti-Flag antibody (Sigma, Germany) at a 1:4000 dilution. Coomassie brilliant blue staining of the Rubisco small subunit (RbcS) protein was used as the loading control. The Western blot assay was performed according to Wang and Cheng [5,48].

## 5. Conclusions

In this study, we identified a DELLA family member, herein named *PpeDGYLA,* in peach, containing a non-canonical DELLA domain with three conserved motifs (DGYLA, LERLE, and AVLYNP). The PpeDGYLA protein did not interact with PpeGID1c and was stable in 35S-PpeDGYLA peach transgenic callus in the absence or presence of GA_3_. The ectopic expression of *PpeDGYLA* in polar and Arabidopsis both showed an extremely dwarfed phenotype, and these transgenic plants were insensitive to GA_3_ treatment. Among the three clasdes of the DELLA family (DELLA-Like, DELLA, and DGLLA), PpeDGYLA clustered into the DGLLA clade and was expressed in all of the analyzed tissues. Altogether, these results contribute to our understanding of the function of PpeDGYLA and offer potential ways to modulate tree size of peach.

## Figures and Tables

**Table 1:** Sequence similarity of DELLA-like amino acid sequences from peach or Arabidopsis.

Identity (%)	PpeDGYLA	PpeDELLA1	PpeDELLA2	AtRGA	AtGAI	AtRGL1	AtRGL2	AtRGL3
PpeDGYLA	-	-	-	-	-	-	-	-
PpeDELLA1	41.82	-	-	-	-	-	-	-
PpeDELLA2	39.94	61.70	-	-	-	-	-	-
AtRGA	42.09	58.99	61.36	-	-	-	-	-
AtGAI	44.96	59.56	61.04	73.59	-	-	-	-
AtRGL1	43.77	58.82	51.42	55.54	58.49	-	-	-
AtRGL2	43.08	60.27	51.19	56.16	55.04	61.27	-	-
AtRGL3	41.64	56.78	50.00	52.71	56.86	59.70	68.95	-

**Table 2:** Analysis of DELLA (DE), DG, and DL sequences in the DELLA proteins of different species.

Phylum	Species	DL	DE	DG	Phylum	Species	DL	DE	DG
Bryophyte	*Marchantia polymorpha*	+			Angiosperm	*Vitis vinifera* Genoscope.12X		+	+
	*Physcomitrella patens*	+				*Linum usitatissimum v1.0*		+	+
	*Sphagnum fallax*	+				*Citrus sinensis v1.1*		+	+
Lycophyte	*Selaginella moellendorffii*	+				*Carica papaya ASGPBv0.4*		+	+
Gymnosperm	*Thuja plicata*		+	+		*Gossypium raimondii v2.1*		+	+
Angiosperm	*Amborella trichopoda*		+	+		*Theobroma cacao v1.1*		+	+
	*Ananas comosus*		+	-		*Cucumis sativus*		+	+
	*Musa acuminata v1*		+	+		*Malus domestica*		+	+
	*Spirodela polyrhiza v2*		+	+		*Pyrus Communis Bartlett*		+	-
	*Zostera marina v2.2*		+	+		*Fragaria vesca v4.0.a2*		+	+
	*Brachypodium distachyon*		+	+		*Prunus persica*		+	+
	*Oryza sativa v7_JGI*		+	+		*Glycine max*		+	+
	*Panicum hallii v2.0*		+	+		*Medicago truncatula*		+	-
	*Setaria italica v2.2*		+	+		*Phaseolus vulgaris*		+	+
	*Sorghum bicolor v3.1.1*		+	+		*Trifolium pratense*		+	+
	*Zea mays Ensembl-18*		+	+		*Arabidopsis thaliana*		+	-
	*Oropetium thomaeum*		+	+		*Boechera stricta v1.2*		+	-
	*Aquilegia coerulea v3.1*		+	+		*Eutrema salsugineum v1.0*		+	-
	*Amaranthus hypochondriacus*		+	+		*Capsella grandiflora v1.1*		+	-
	*Daucus carota*		+	+		*Populus trichocarpa v4.0*		+	-
	*Mimulus guttatus*		+	-		*Salix purpurea v1.0*		+	-
	*Solanum lycopersicum* iTAG2.4		+	-		*Manihot esculenta v6.1*		+	-
	*Eucalyptus grandis v2.0*		+	+		*Ricinus communis v0.1*		+	+

Note: The red font in the table is the DELLA (DE), DG, and DL sequences in the peach.

## Data Availability

Not applicable.

## Supplementary Materials

The following supporting information can be downloaded at: https://www.mdpi.com/article/10.3390/ijms24076789/s1.
