# Supplementary material for: Peach DELLA Protein PpeDGYLA Is Not Degraded in the Presence of Active GA and Causes Dwarfism When Overexpressed in Poplar and Arabidopsis
Source: Int J Mol Sci. 2023 Apr 6;24(7):6789. doi: 10.3390/ijms24076789 (PMC10095214; doi:10.3390/ijms24076789)
Supplement: Supplementary file 1 [file ijms-24-06789-s001.zip › ijms-2309464-supplementary.pdf]

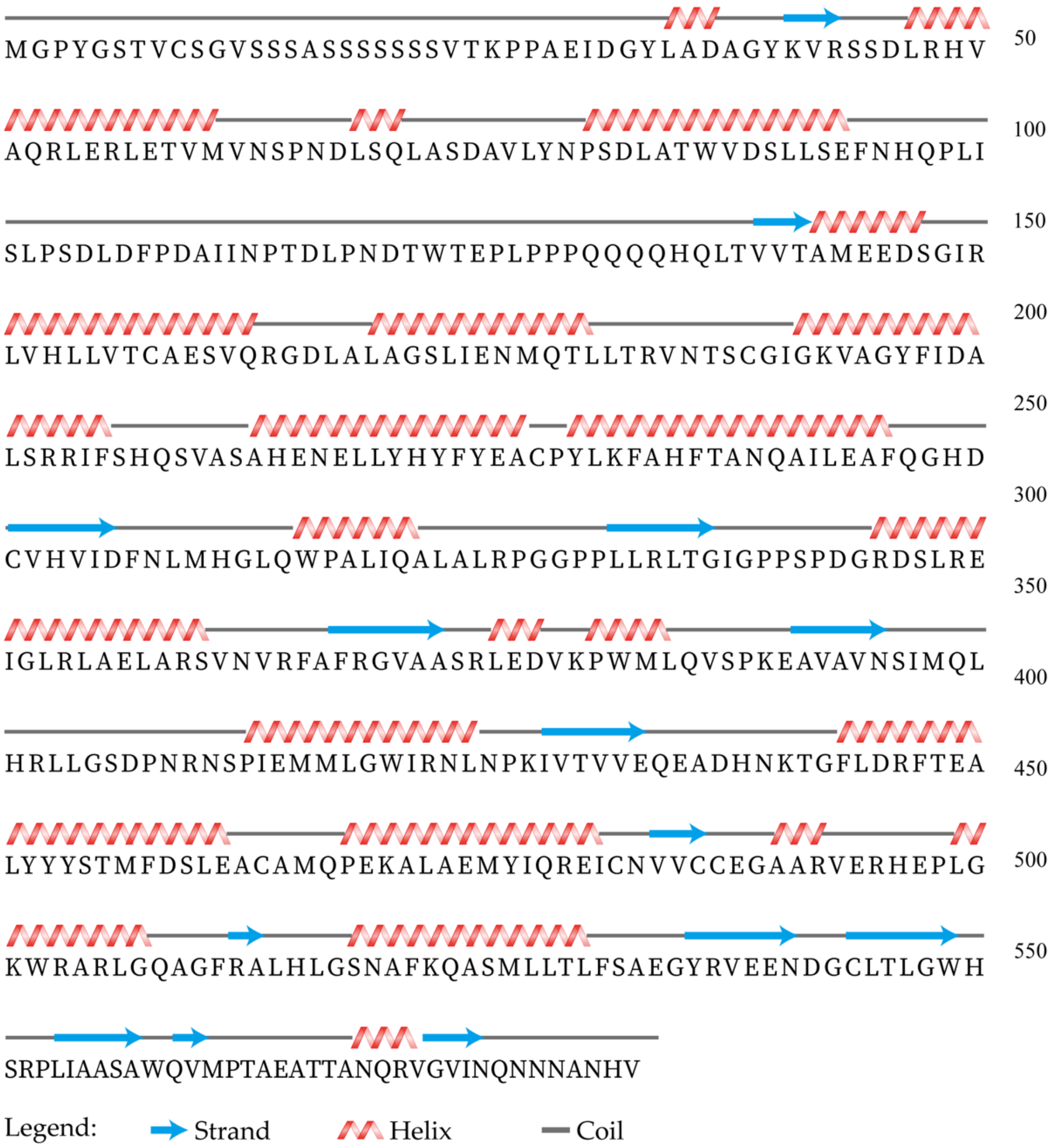

Figure S1 Predicted secondary structure of PpeDGYLA. Predictions were made using PSIPRED and GORIV.

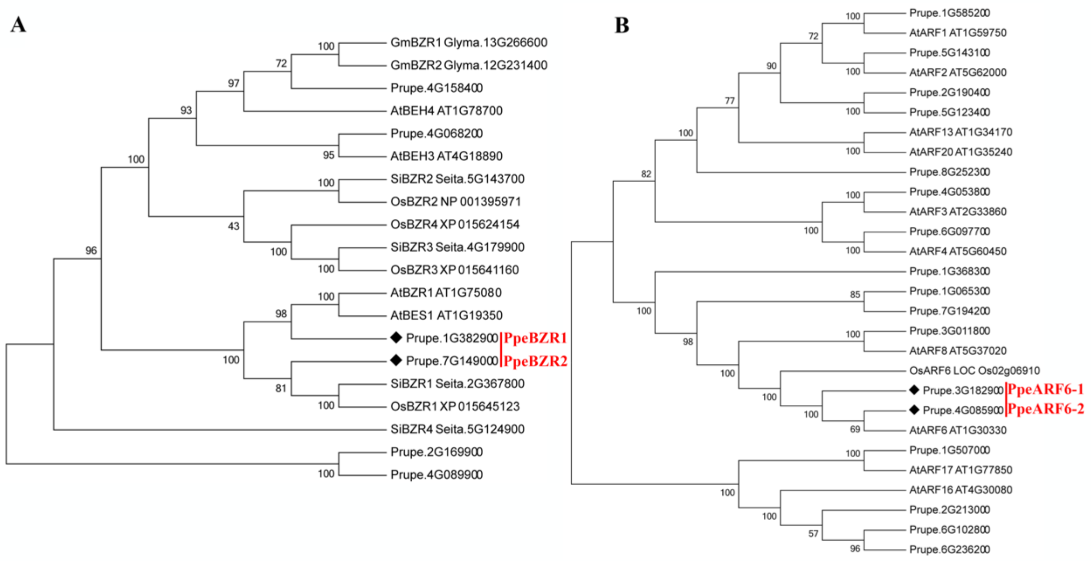

Figure S2 Phylogenetic tree of *BZR* (A) and *ARF* (B) transcription factor genes in the peach genome and the orthologous genes from other species, including *Glycine max*, *Arabidopsis thaliana*, *Oryza sativa* and *Setaria italica*. Accession numbers are listed after the gene name. The orthologous genes from peach of *BZR* and *ARF* analyzed in this study were in red.

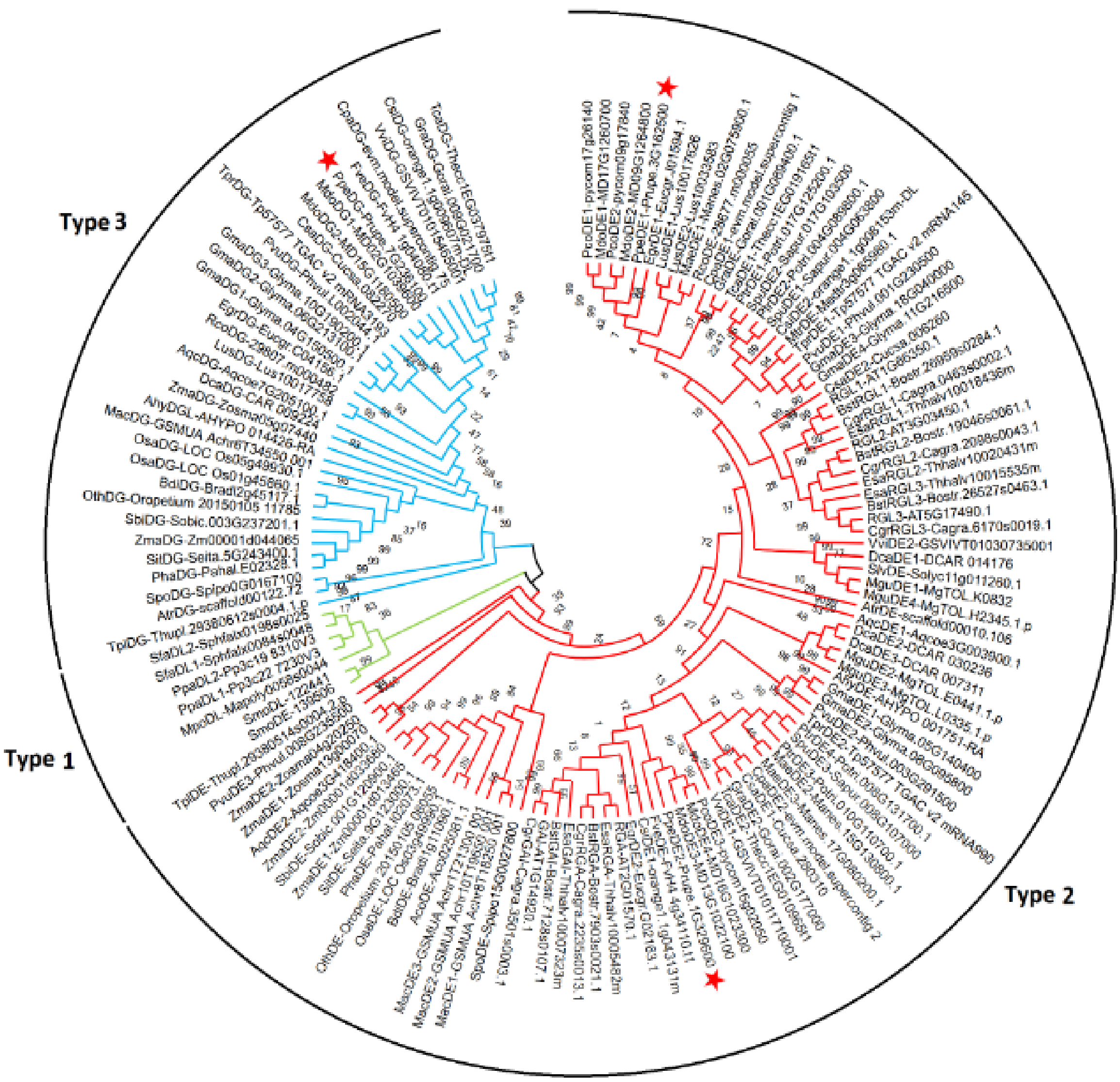

Figure S3 Phylogenetic tree of DELLA-like, DELLA and DGLLA proteins in land plant taxa constructed by neighbor-joining method. Type 1 indicates DELLA members from bryophytes and lycophytes and are named as DELLA-Like (DL). Type 2 indicates DELLA members from gymnosperms and angiosperms and which contain a canonical DELLA domain starting with DELLA (DE). Type 3 indicates DELLA proteins from gymnosperms and angiosperms and which started with a DGLLA (DG) DELLA domain. Red stars indicated the DELLA members in peach.

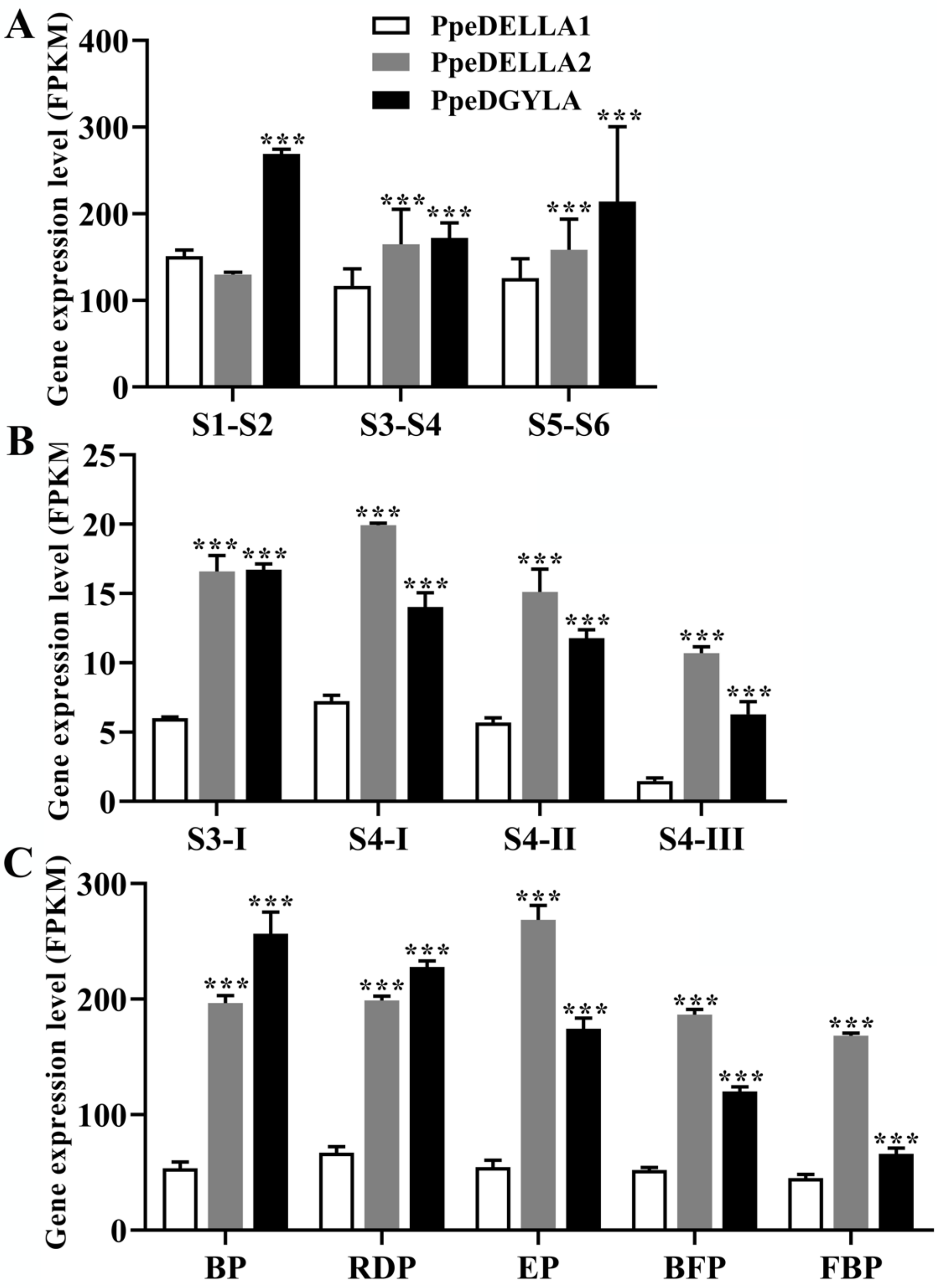

Figure S4 The transcript levels of the three peach *DELLA* genes based on RNA-seq transcriptome data. (A) In stem internodes, where internodes are numbered S1-S6 from top to bottom of stems, S1-S2, S3-S4 and S5-S6 represent combined samples of the internodes S1 and S2, S3 and S4, and S5 and S6. (B) In fruit, where S3-I, S4-I, S4-II, and S4-III represent 118, 120, 122 and 124 d after full bloom, respectively. (C) In flower, where BP: bud period; RDP: red dot period; EP: equivalent in size between petal and sepal period; BFP: budding flower period; FBP: full bloom period. *** indicate significant difference at $P < 0.001$

Table S1 The primers of PpPIFs for qRT-PCR and vector construction

Name	Primer Sequences (5'-3')
PpeDELLA1-qF	TGTGGAGCAGGAGGCAAAC
PpeDELLA1-qR	TGACTCGGACCGGACGAT
PpeDELLA2-qF	GCAAACCACAACGGTCCAGT

PpeDELLA2-qR	GCTCTGACATCGCCTTATCCT
PpeDGYLA-qF	TTAGGGCTCTGCATTTGGGTT
PpeDGYLA-qR	GGTTGGCTGTGGTTGCTTCA
PpeDELLA1(pSAK277-Flag)-F	TCCAAAGAATTCCCCGGTACCATGAAGAGAGATCACCGCG
PpeDELLA1(pSAK277-Flag)-R	ATGATCTTTGTAATCCTCGAGCCGGGTTGACTCAGTCGAA
PpeDGYLA(pSAK277-Flag)-F	TCCAAAGAATTCCCCGGTACCATGGGGCCCTACGGTTCAA
PpeDGYLA(pSAK277-Flag)-R	ATGATCTTTGTAATCCTCGAGGACATGATTGGCATTATTA
PpePIF8(GAL4AD)-F	CAGATTACGCTCATATGATGAACCAGTGTGTTCCCAGC
PpePIF8(GAL4AD)-R	ACGATTCATCTGCAGCTCGAGGCTCTTAGAACTAGATGC
PpeDELLA1(GAL4AD)-F	CAGATTACGCTCATATGATGAAGAGAGATCACCGCGG
PpeDELLA1(GAL4AD)-R	CACCCGGGTGGAATTCTCACCGGGTTGACTCAGTCG
PpeDGYLA(GAL4AD)-F	GTACCAGATTACGCTCATATGATGGGGCCCTACGGTTCA
PpeDGYLA(GAL4AD)-R	ACGATTCATCTGCAGCTCGAGTTAGACATGATTGGCATTATTATT
PpeGID1c(GAL4BD)-F	TCAGAGGAGGACCTGCATATGATGGCTGGGACCAACGAAGT
PpeGID1c(GAL4BD)-R	TTATGCGGCCGCTGCAGACCGCACGCGATGAAAGTC
PpeDGYLA(GAL4BD)-F	TCAGAGGAGGACCTGCATATGATGGGGCCCTACGGTTCA
PpeDGYLA(GAL4BD)-R	TCGACGGATCCCCGGGAATTCTTAGACATGATTGGCATTATTATT
PpeBZR1(GAL4AD)-F	GGATCCCCGGGAATTCGAGAGGGAAAAGAGAGGGAAGAT
PpeBZR1(GAL4AD)-R	ATGCCCACCCGGGTGGAATTCCTTAACTTCGAGGCTTACCACTT
PpeBZR2(GAL4AD)-F	GCCATGGAGGCCAGTGAATTCGCTCAAAGGGTAAGCAGGTGG
PpeBZR2(GAL4AD)-R	ATGCCCACCCGGGTGGAATTCCGTCCCTCCTGGTTATACACATT
PpeARF6-1(GAL4AD)-F	CAGATTACGCTCATATGATGAGACTTTCGTCTTCATC
PpeARF6-1(GAL4AD)-R	CATCTGCAGCTCGAGTTAGTAGTCCAGTGAGCC
PpeARF6-2(GAL4AD)-F	CAGATTACGCTCATATGAGGCTCTCATCTGCTGGTTTTAG
PpeARF6-2(GAL4AD)-R	TCATCTGCAGCTCGAGGGGTTGGAGGAGATGCAAATAT

Table S2 Physical and chemical properties of the PpeDGYLA

**Characteristics**	**Predictive values**
Number of amino acids	537
Molecular weight	59.16 KDa
Theoretical pI	5.58
The instability index (II)	43.68
Aliphatic index	90.86

Grand average of hydropathicity (GRAVY)	-0.138
Alpha helix	46.00 %
Extended strand	12.48 %
Random coil	41.53 %
